# Interpersonal violence attributed to smoking, globally and in Iran: Global burden of disease study

**DOI:** 10.1097/MD.0000000000042983

**Published:** 2025-08-08

**Authors:** Moien A.B. Khan, Sohrab Amiri

**Affiliations:** a Department of Family Medicine, College of Medicine and Health Sciences, United Arab Emirates University, Al-Ain, United Arab Emirates; b Spiritual Health Research Center, LifeStyle Institute, Baqiyatallah University of Medical Sciences, Tehran, Iran.

**Keywords:** global burden of disease, interpersonal violence, prevalence, smoking

## Abstract

Despite the effects of smoking on health have been extensively studied, the studies on smoking and its effects on interpersonal violence are very few. This research aims to investigate the burden of interpersonal violence attributed to smoking in Iran and compare it with the global. In global burden of disease 2021, the relationship between 88 risk factors with selected health outcomes has been estimated. Summary exposure value (SEV) is the RR-weighted prevalence of exposure, a univariate measure of risk weighted exposure. We report SEVs on a scale from 0% to 100% on which a decline in SEV indicates reduced exposure to a given risk factor and an increase in SEV indicates increased exposure. All age and age-standardized estimates of interpersonal violence attributed to smoking were calculated for disability-adjusted life years, years lived with disability, and years of life lost, and death for the years 1990 to 2021. The global number of disability-adjusted life years cases of interpersonal violence attributed to smoking was 16,260.52 per 1,00,000 in 2021 (95% uncertainty interval 12,268.37–20,718.62). The number of disability-adjusted life years cases of interpersonal violence attributed to smoking in Iran was 142.97 per 1,00,000 (95% uncertainty interval 102.78–185.83). Age-standardized disability-adjusted life years rate per 1,00,000 globally was 0.19 (95% uncertainty interval 0.14–0.24), and in Iran was 0.15 (95% uncertainty interval 0.11–0.19) in 2021. The summary of this research shows that the burden of interpersonal violence attributed to smoking is significant both at the global and national levels, although the prevalence of smoking has decreased in recent decades, it is necessary to implement health policies to reduce the damage caused by smoking.

## 1. Introduction

Over the past decades, smoking has placed a significant burden on the health system, with more than 200 million deaths attributable to smoking and the annual cost of smoking has increased to over $1 trillion.^[1,2]^ According to the published report of the most comprehensive epidemiological study, 1.14 billion people in the world are current smokers, who have smoked a total of 7.41 trillion cigarette-equivalents of tobacco.^[3]^ Although compared to 1990, the amount of smoking in the world has decreased by 27.5% in men and 37.7% in women, the growth of the world population has significantly increased the number of smokers.^[3]^ The estimation of the prevalence of smoking in 2020 in adults has shown that this prevalence was 32.6% in men and 6.5% in women.^[4]^

Smoking is responsible for 769 million (716–820) deaths and 200 million (185–214) disability-adjusted life years (DALYs).^[3]^ Cohort studies have shown that at least 50% of long-term smokers will die from causes directly attributable to smoking and on average life expectancy in these people is 10 years less than those who have never smoked.^[5,6]^ Smoking is associated with a range of devastating physical health problems and increases the risk of disease including cancer,^[7]^ metabolic syndrome,^[8]^ fracture,^[9]^ stroke,^[10]^ diabetes,^[11]^ physical impairment,^[12]^ self-rated haelth,^[13]^ sleep,^[14]^ and mental disorders.^[15]^ Determining the effects of smoking on physical and mental health has been widely considered since the 1960s.^[16,17]^ The impact of smoking on mental health has been established, so that smoking is known as a risk factor for schizophrenia,^[18]^ bipolar disorder,^[19]^ and major depression.^[20,21]^ One of the consequences that have been expressed concerning smoking is interpersonal violence.^[22]^

The World Health Organization recognizes violence as “The intentional use of physical force or power, threatened or actual, against oneself, another person, or against a group or community that either results in or has a high likelihood of resulting in injury, death, psychological harm, mal-development, or deprivation.”^[23]^ Interpersonal violence is defined as “ intentional use of physical force or power against other people by an individual or small group of individuals. Interpersonal violence may be physical, sexual, or psychological (also called emotional violence), and it may involve deprivation and neglect.”^[24]^ Interpersonal violence is an important issue in health and according to the global burden of diseases 2019, 310 million cases of interpersonal violence occurred in the world and 415 thousand people lost their lives due to interpersonal violence.^[25,26]^

Despite extensive research on the health consequences of smoking, including its effects on physical health problems such as cancer, metabolic syndrome, and mental disorders, there remains a significant gap in understanding the relationship between smoking and interpersonal violence. Research suggests a potential link between smoking and violence.^[27]^ A community based study Lewis et al found that smoking is associated with increased aggression, possibly due to nicotine’s effects on impulse control.^[22]^ Gehricke et al demonstrated that nicotine exposure may heighten aggressive responses.^[28]^ Epidemiological studies further indicate that smoking is associated with higher rates of interpersonal violence, even after controlling for confounding factors such as socioeconomic status and mental health.^[27,29]^ However, the mechanisms underlying this association are complex and may involve both direct neurobiological effects and indirect social or psychological pathways.^[28]^ Despite these insights, existing research often lacks generalizability due to limited sample sizes or geographic scope, highlighting the need for population-level analyses. This research aims to fill this gap by investigating the burden of interpersonal violence attributed to smoking both globally and in Iran. By evaluating DALYs, years lived with disability (YLDs), years of life lost (YLLs), and deaths attributed to interpersonal violence linked to smoking, based on the Global Burden of Disease 2021 study, this research provides insights that could inform public health policies aimed at reducing both smoking prevalence and associated violent behaviors.

## 2. Materials and methods

### 2.1. Ethical review

This is a secondary study, no new data were collected, and ethical approval is not required.

### 2.2. Involving human participants and/or animals

None applicable.

### 2.3. Informed consent

None applicable.

### 2.4. Data source

For this research, global burden of disease 2021^[30–32]^ was used. GBD 2021 has been reporting incidence, prevalence, DALYs, YLDs, YLLs, and death for 371 diseases and injuries along with estimates of healthy life expectancy. These estimates are provided for sex, and age groups and for 204 countries and territories, including subnational estimates for 21 countries.^[31]^ Data sources used in GBD 2021 included 1,00,983 data sources (19,189 new data sources for DALYs) 12 new causes, and other important methodological updates.^[31]^ Inclusion criteria were cases of interpersonal violence directly attributed to smoking, as defined by intentional physical force from another individual, excluding military or police-related incidents. Exclusion criteria encompassed nonsmoking-related violence and cases where the link to smoking was unclear. More details about the data sources, and methodology of GBD 2021 are reported elsewhere.^[31]^

### 2.5. Case definitions

Tobacco is defined as tobacco smoking, chewing tobacco use, and secondhand smoke exposure.^[33]^ Interpersonal violence is defined as “Death or disability from intentional use of physical force or power, threatened or actual, from another person or group not including military or police forces” (International Classification of Diseases [ICD] 10; T74.2-T76.22, X85-Y08.9, Y87.1-Y87.2). Physical violence by firearm is defined as “Death or disability from intentional use of a firearm by another person, not including military or police forces” (ICD 10; X93-X95.9). Physical violence by sharp object is defined as “Death or disability from intentional use of physical force or power by a sharp object from another person, not including military or police forces” (ICD 10; X99-X99.9). Physical violence by other means is defined as “Death or disability from intentional use of physical force or power by an object other than a firearm or sharp object from another person not including legal, military, or police forces” (ICD 10; T74.2-T76.22, Y05-Y05.9). Sexual violence is defined as “the proportion of the population that experienced at least 1 event of sexual violence in the last year. We define sexual violence as any sexual assault, including both penetrative sexual violence (rape) and non-penetrative sexual violence (other forms of unwanted sexual touching” [ICD 10; X85-X92.9, X96-X98.9, Y00-Y04.9, Y06-Y08.9, Y87.1-Y87.2]).^[31]^ Refer elsewhere for more details on GBD methodology.^[31]^

### 2.6. Estimation framework

YLDs were estimated by multiplying prevalence estimates at varying levels of severity by an appropriate disability weight.^[31]^ YLLs were calculated by multiplying cause-specific deaths by the years of life expected to remain at death based on a normative life expectancy.^[31]^ DALYs were calculated as the sum of YLDs and YLLs.^[31]^

### 2.7. Statistics

In GBD 2021, the relationship between 88 risk factors with selected health outcomes has been estimated.^[30]^ SEV is the RR-weighted prevalence of exposure, a univariate measure of risk weighted exposure, taking the value zero when no excess risk for a population exists and the value 1 when the population is at the highest level of risk. We report SEVs on a scale from 0% to 100% on which a decline in SEV indicates reduced exposure to a given risk factor and an increase in SEV indicates increased exposure.^[30]^ All age and Age-standardized estimates of interpersonal violence attributed to smoking were calculated for DALYs, YLDs, and YLLs, and death for the years 1990 to 2021.^[31]^ Each of the disease burden indicators was examined in the period of 1990 to 2021. Estimates were based on per 1,00,000 populations. The 95% uncertainty interval was reported for each of the reported estimates. More details about data, data processing, and modeling are elsewhere which are related to GBD 2021.^[31]^ GBD 2021 complies with the guidelines for accurate and transparent health estimates reporting^[34]^; Analyses were completed using Python (version 3.10.4), Stata (version 13.1), and R (version 4.2.1).

## 3. Results

### 3.1. Summary exposure value for smoking in Iran and global

**Figure 1.**
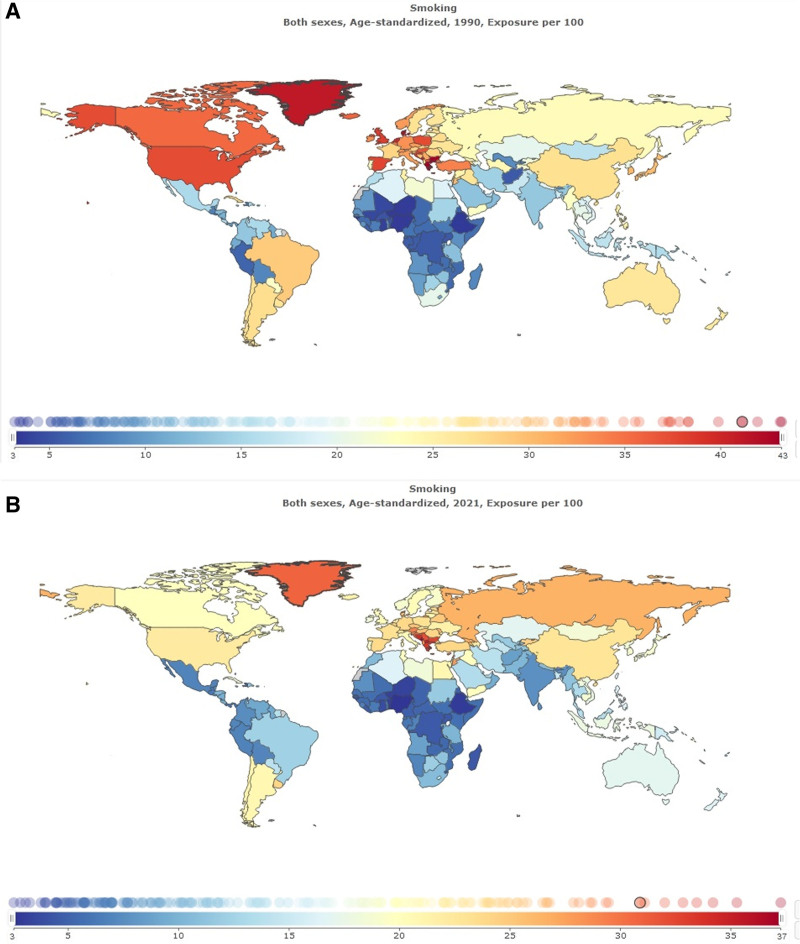
Summary exposure value for smoking: (A) both sexes, age-standarsized, 1990, exposure per 100; (B) both sexes, age-standarsized, 2021, exposure per 100.

**Figure 2.**
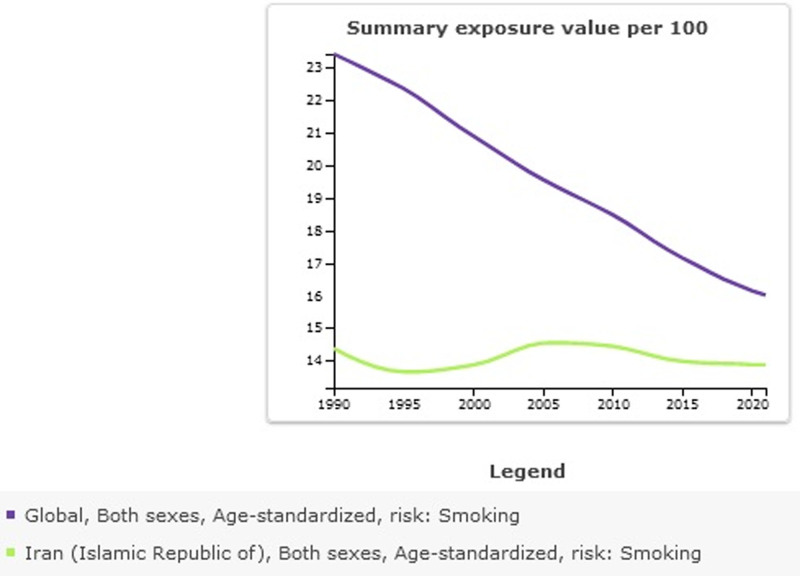
Summary exposure value (per 100) for smoking.

In Table 1 summary exposure value (SEV) for smoking in global and Iran is shown. Age-standardized SEV per 100 in Iran was 13.87 (95% uncertainty interval 12.72–15.15). Percentage change from 1990 to 2021 in Iran was −0.03 (95% uncertainty interval −0.15 to 0.09); this shows the decline of smoking in Iran during the last 3 decades. Age-standardized SEV per 100 in global was 16.01 (95% uncertainty interval 15.57–16.47; Fig. 1). Percentage change from 1990 to 2021 in Iran was −0.32 (95% uncertainty interval −0.33 to −0.3); this shows the reduction of smoking in the global has been more compared to Iran (Fig. 2).

**Table 1:** All age and age-standardized rate summary exposure value for smoking between 1990 and 2021 in Iran and global.

Measure	Location	1990			2021			Percentage change 1990–2021		
		Value	Lower	Upper	Value	Lower	Upper	Value	Lower	Upper
All ages summary exposure value	Global	23.77	23.26	24.33	16.08	15.65	16.55	−0.32	−0.34	−0.31
Age-standardized summary exposure value	Global	23.42	22.91	24	16.01	15.57	16.47	−0.32	−0.33	−0.3
All ages summary exposure value	Iran	14.84	13.45	16.38	14.31	13.04	15.7	−0.04	−0.15	0.1
Age-standardized summary exposure value	Iran	14.38	13.06	15.79	13.87	12.72	15.15	−0.03	−0.15	0.09

### 3.2. Summary exposure value for smoking in males and females

**Figure 3.**
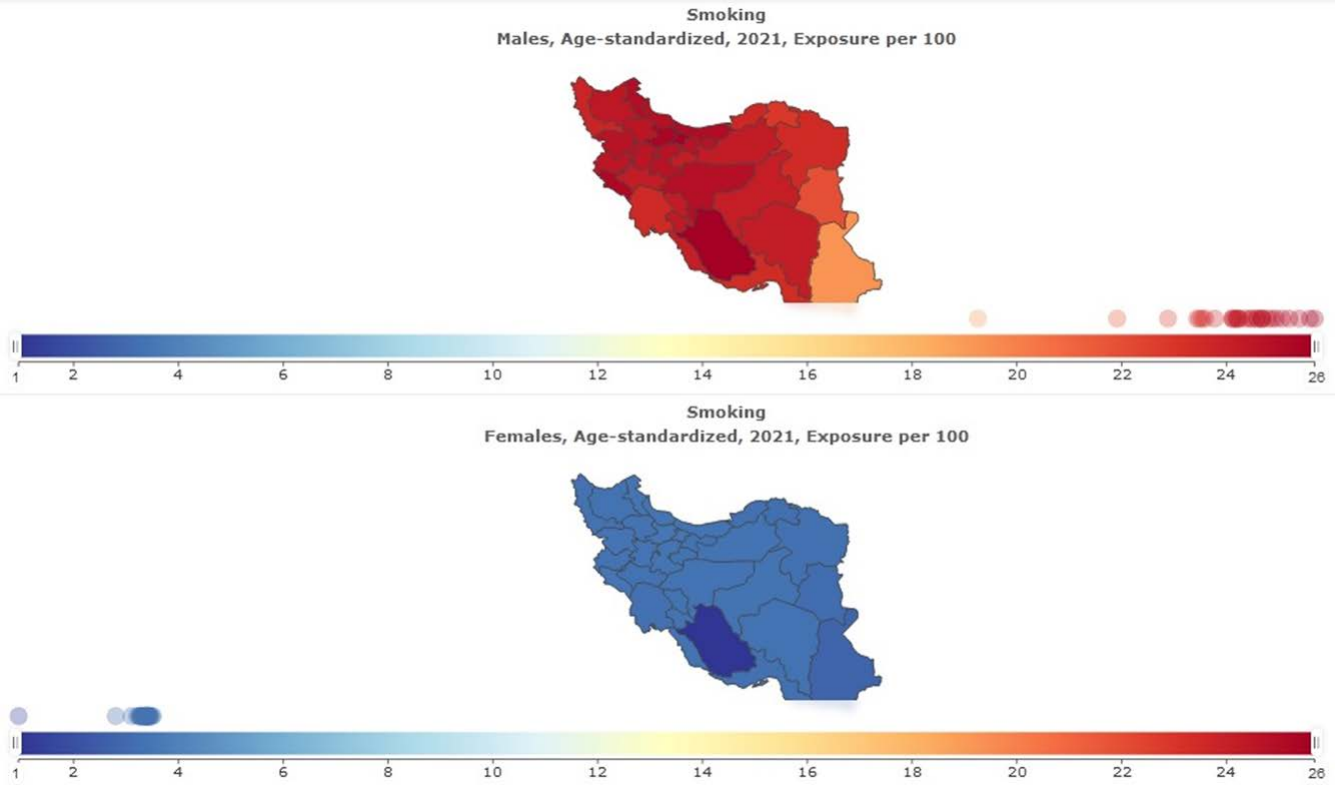
Summary exposure value for smoking in male and female.

**Figure 4.**
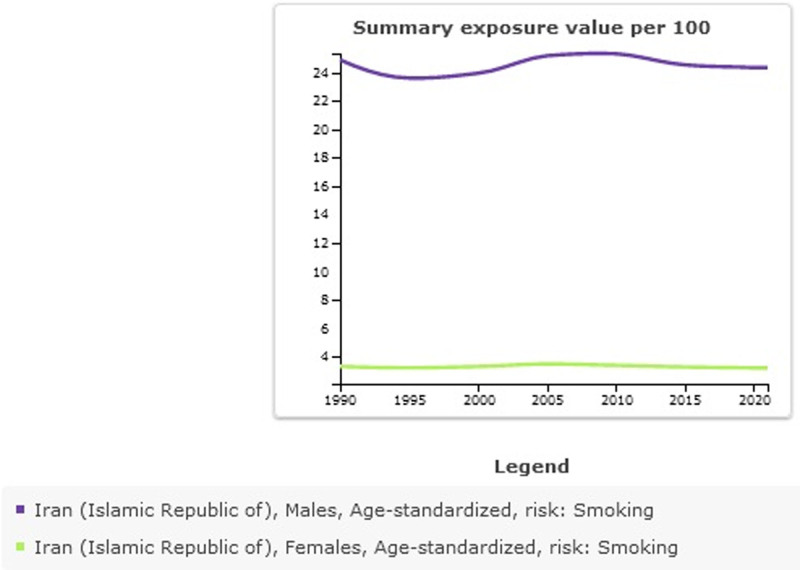
Summary exposure value for smoking in male and female 1990–2021.

In Table 2 and Figure 3 SEV for Smoking in Iran in males and females is shown. Age-standardized SEV per 100 in males was 24.34 (95% uncertainty interval 22.17–26.59). Percentage change from 1990 to 2021 in Iran was −0.02 (95% uncertainty interval −0.14 to 0.11); this shows the decline of smoking in males. Age-standardized SEV per 100 in females was 3.19 (95% uncertainty interval 2.58–3.94). Percentage change from 1990 to 2021 in Iran was −0.04 (95% uncertainty interval −0.29 to 0.28); this shows the decline of smoking in females. The findings of men and women show that the exposure to smoking in males in 2021 is almost 8 times that of females (Fig. 4).

**Table 2:** All age and age-standardized rate summary exposure value for smoking between 1990 and 2021 in Iran for male and female.

Measure	Sex	Year								
		1990			2021			Percentage change 1990–2021		
		Value	Lower	Upper	Value	Lower	Upper	Value	Lower	Upper
All ages summary exposure value	Males	25.71	23.21	28.48	25.19	23.01	27.59	−0.02	−0.14	0.12
	Females	3.33	2.6	4.21	3.21	2.59	3.99	−0.04	−0.3	0.29
Age-standardized summary exposure value	Males	24.89	22.58	27.32	24.34	22.17	26.59	−0.02	−0.14	0.11
	Females	3.31	2.61	4.17	3.19	2.58	3.94	−0.04	−0.29	0.28

### 3.3. Summary exposure value for smoking in provinces

**Figure 5.**
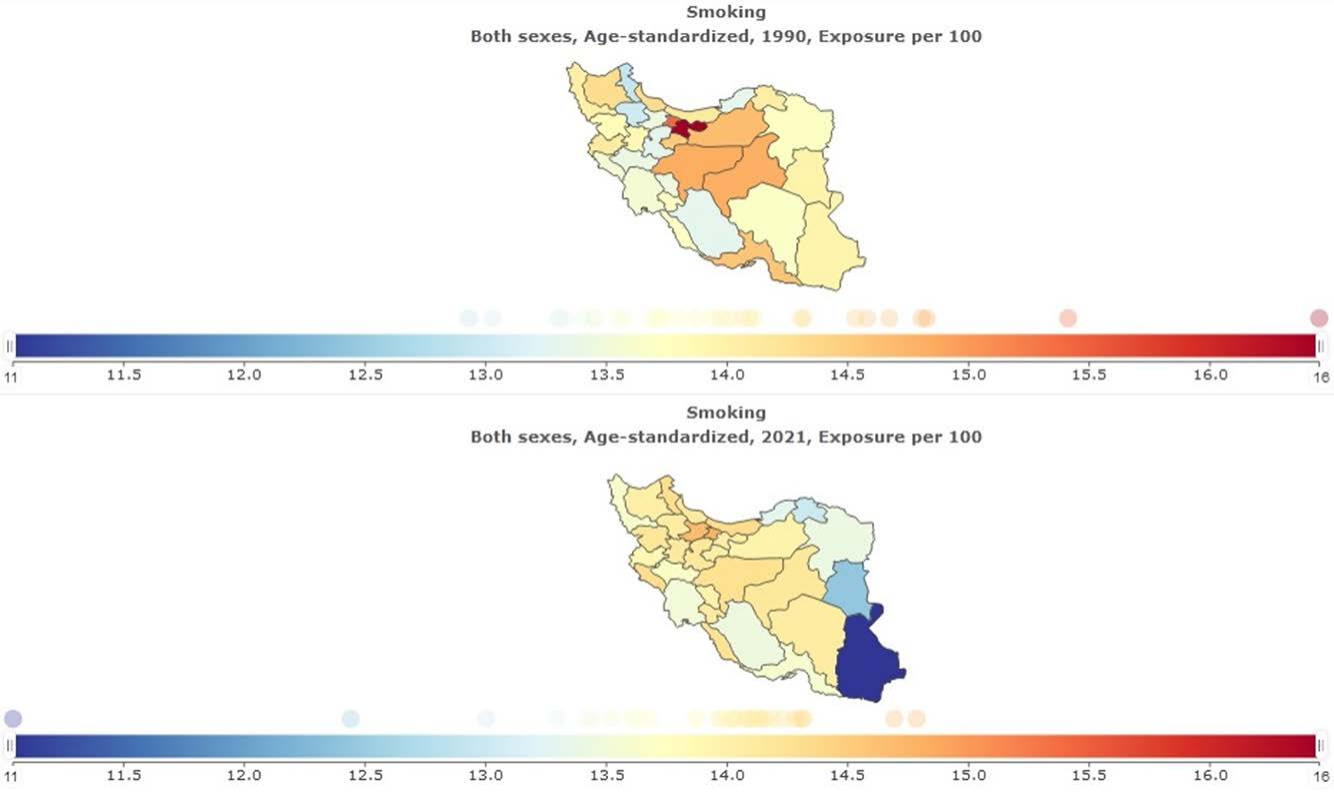
Summary exposure value for smoking in provinces.

In Table 3 and Figure 5, SEV for Smoking in provinces is shown. The highest SEV in 1990 was for Tehran 16.45 (95% uncertainty interval 14.56–18.44); the lowest was for Ardebil 12.93 (95% uncertainty interval 11.17–15.02). The highest SEV in 2021 was for Alborz 14.78 (95% uncertainty interval 13.01–16.73); the lowest was for Sistan and Baluchistan 11.04 (95% uncertainty interval 9.54–12.71). The highest percentage change from 1990 to 2021 was in Sistan and Baluchistan −0.21 (95% uncertainty interval −0.33 to −0.06).

**Table 3:** Age-standardized rate summary exposure value for smoking between 1990 and 2021 in provinces.

Location	Year					
	1990			2021		
	Value	Lower	Upper	Value	Lower	Upper
Alborz	15.41	13.44	17.5	14.78	13.01	16.73
Ardebil	12.93	11.17	15.02	14.3	12.49	16.17
Bushehr	13.71	11.83	15.8	14.23	12.39	16.3
Chahar Mahaal and Bakhtiari	13.45	11.6	15.29	13.96	12.31	15.86
East Azarbayejan	14.32	12.5	16.17	14.03	12.4	15.85
Fars	13.3	11.48	15.33	13.45	11.82	15.05
Gilan	14.31	12.34	16.43	14.13	12.35	15.98
Golestan	13.32	11.49	15.08	13.3	11.71	15.09
Hamadan	13.87	12.02	15.93	14.12	12.51	16.08
Hormozgan	14.58	12.75	16.53	13.61	11.82	15.44
Ilam	13.57	11.67	15.4	14.31	12.43	16.18
Isfahan	14.83	12.83	16.86	14.28	12.58	16
Kerman	13.68	11.99	15.57	14.08	12.36	16.27
Kermanshah	14.09	12.18	16.13	13.87	12.36	15.44
Khorasan-e-Razavi	13.71	11.86	15.63	13.42	11.74	15.12
Khuzestan	13.55	11.83	15.71	13.52	11.74	15.36
Kohgiluyeh and Boyer-Ahmad	13.74	11.82	15.81	14.1	12.36	15.9
Kurdistan	13.8	11.87	15.77	14.19	12.34	16.2
Lorestan	13.4	11.56	15.51	13.68	11.79	15.53
Markazi	13.3	11.41	15.1	14.15	12.31	16.02
Mazandaran	14.11	12.31	16.06	14.31	12.69	16.07
North Khorasan	14.06	12.33	15.92	13	11.31	14.92
Qazvin	13.45	11.62	15.24	14.69	12.85	16.66
Qom	14.53	12.77	16.67	14.02	12.22	15.97
Semnan	14.67	12.81	16.65	13.98	12.17	15.99
Sistan and Baluchistan	13.94	11.98	15.81	11.04	9.54	12.71
South Khorasan	13.98	12.14	15.8	12.44	10.74	14.3
Tehran	16.45	14.56	18.44	14.04	12.41	15.69
West Azarbayejan	14.01	12.26	15.98	13.63	11.86	15.39
Yazd	14.81	12.86	16.93	14.15	12.34	16.23
Zanjan	13.03	11.23	14.84	14.08	12.36	16.06

### 3.4. Interpersonal violence attributed to smoking

**Figure 6.**
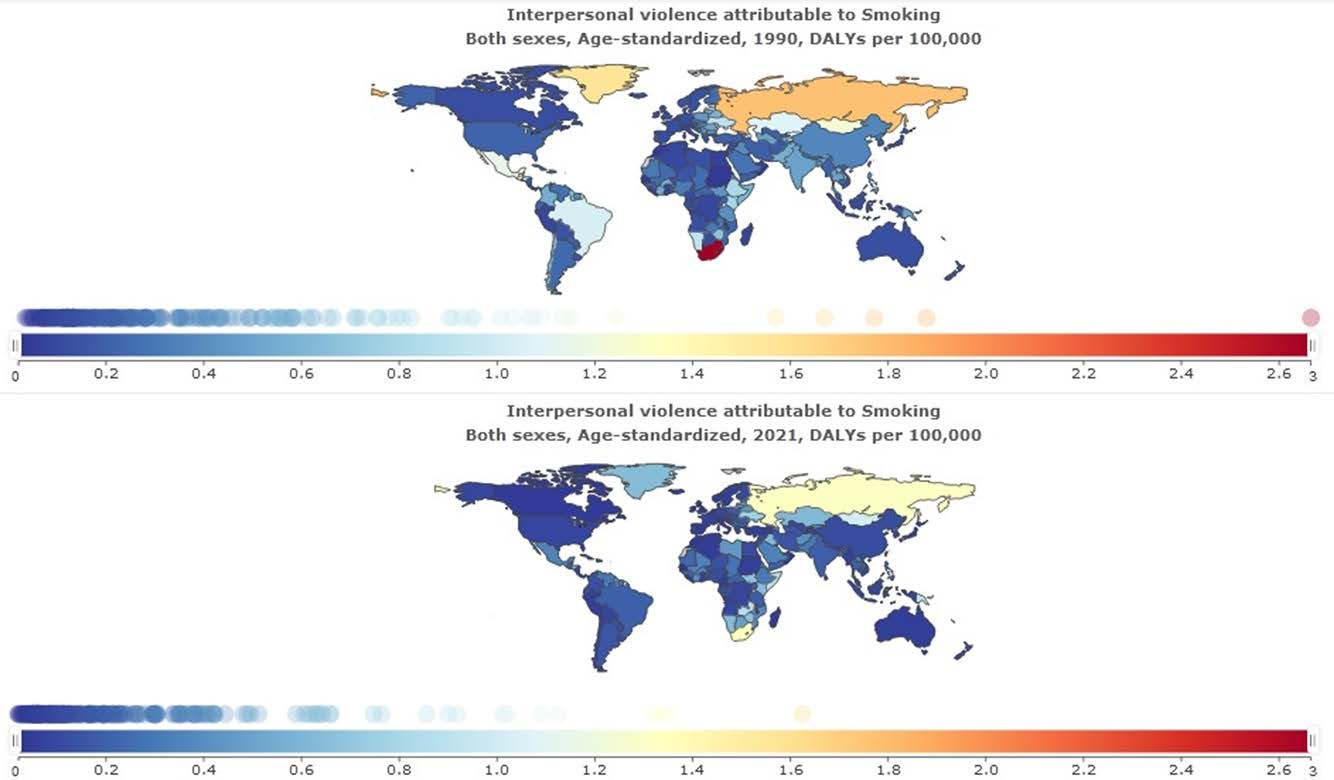
Interpersonal violence attributed to smoking.

**Figure 7.**
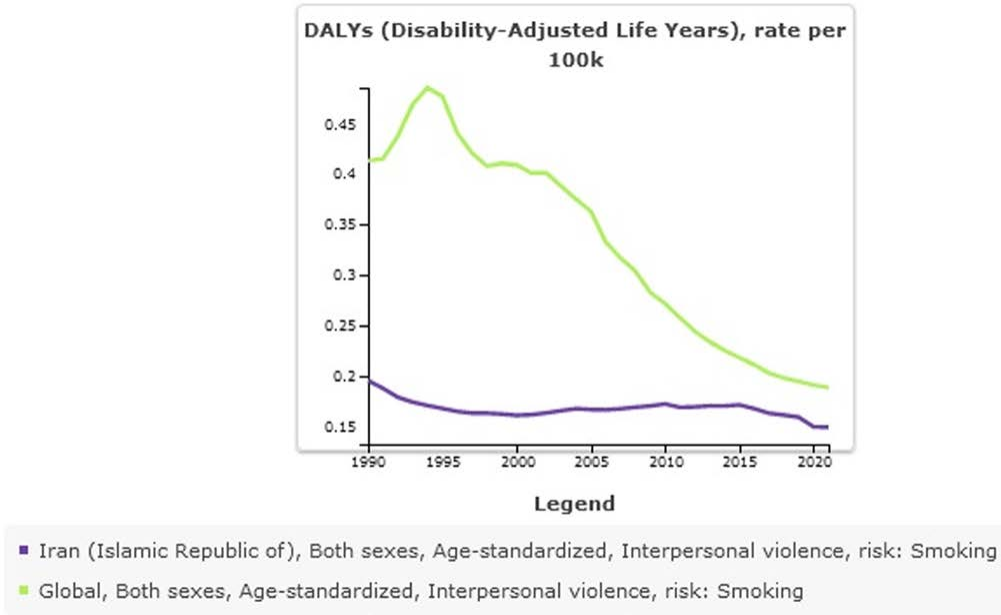
Interpersonal violence attributed to smoking 1990–2021.

The burden of interpersonal violence attributed to smoking is reported in Table 4 and Figures 6–7. The global number of DALY cases of interpersonal violence attributed to smoking was 16,260.52 per 1,00,000 in 2021 (95% uncertainty interval 12,268.37–20,718.62). The number of DALY cases of interpersonal violence attributed to smoking in Iran was 142.97 per 1,00,000 (95% uncertainty interval 102.78–185.83). Age-standardized DALYs rate per 1,00,000 globally was 0.19 (95% uncertainty interval 0.14–0.24), and in Iran was 0.15 (95% uncertainty interval 0.11– 0.19) in 2021.

**Table 4:** Interpersonal violence (age-standardized DALYs, YLDs, YLLs, and deaths) attributed to smoking between 1990 and 2021 (per 1,00,000).

Measure	Metric	Location	Age	Year					
				1990			2021		
				Value	Lower	Upper	Value	Lower	Upper
DALYs (disability-adjusted life years)	Number	Iran	All ages	61.58	44.04	82.06	142.97	102.78	185.83
	Percent		All ages	0.05	0.04	0.06	0.09	0.06	0.11
	Rate		All ages	0.11	0.08	0.14	0.17	0.12	0.22
	Percent		Age-standardized	0.09	0.06	0.11	0.08	0.06	0.1
	Rate		Age-standardized	0.2	0.14	0.26	0.15	0.11	0.19
	Number	Global	All ages	17,530.86	13,299.32	22,038.81	16,260.52	12,268.37	20,718.62
	Percent		All ages	0.07	0.05	0.08	0.06	0.05	0.08
	Rate		All ages	0.33	0.25	0.41	0.21	0.16	0.26
	Percent		Age-standardized	0.09	0.07	0.11	0.06	0.04	0.07
	Rate		Age-standardized	0.41	0.31	0.52	0.19	0.14	0.24
YLDs (years lived with disability)	Number	Iran	All ages	23.65	15.48	34.66	52.73	34.93	76.27
	Percent		All ages	0.07	0.05	0.1	0.1	0.07	0.14
	Rate		All ages	0.04	0.03	0.06	0.06	0.04	0.09
	Percent		Age-standardized	0.12	0.08	0.15	0.11	0.07	0.14
	Rate		Age-standardized	0.08	0.05	0.11	0.06	0.04	0.08
	Number	Global	All ages	7309.23	4990.02	10,308.02	6453.89	4406.82	9237.22
	Percent		All ages	0.16	0.12	0.2	0.12	0.09	0.16
	Rate		All ages	0.14	0.09	0.19	0.08	0.06	0.12
	Percent		Age-standardized	0.2	0.14	0.24	0.12	0.08	0.15
	Rate		Age-standardized	0.18	0.12	0.25	0.07	0.05	0.11
YLLs (years of life lost)	Number	Iran	All ages	37.94	26.04	51.52	90.24	64.88	116.81
	Percent		All ages	0.04	0.03	0.05	0.08	0.06	0.1
	Rate		All ages	0.07	0.05	0.09	0.11	0.08	0.14
	Percent		Age-standardized	0.07	0.05	0.1	0.07	0.05	0.09
	Rate		Age-standardized	0.12	0.08	0.16	0.09	0.07	0.12
	Number	Global	All ages	10,221.63	8034.09	12,647.72	9806.63	7533.73	12,072.67
	Percent		All ages	0.05	0.04	0.06	0.05	0.04	0.06
	Rate		All ages	0.19	0.15	0.24	0.12	0.1	0.15
	Percent		Age-standardized	0.06	0.05	0.08	0.04	0.03	0.05
	Rate		Age-standardized	0.24	0.19	0.29	0.11	0.09	0.14
Deaths	Number	Iran	All ages	1	0.7	1.38	2.34	1.68	3.03
	Percent		All ages	0.07	0.05	0.09	0.11	0.08	0.14
	Rate		All ages	0	0	0	0	0	0
	Percent		Age-standardized	0.11	0.08	0.15	0.11	0.08	0.13
	Rate		Age-standardized	0	0	0	0	0	0
	Number	Global	All ages	290.69	227.46	362.31	287.65	221.27	353.01
	Percent		All ages	0.08	0.06	0.1	0.07	0.06	0.09
	Rate		All ages	0.01	0	0.01	0	0	0
	Percent		Age-standardized	0.1	0.08	0.13	0.07	0.05	0.08
	Rate		Age-standardized	0.01	0.01	0.01	0	0	0

DALYs = disability-adjusted life years, YLDs = years lived with a disability, YLLs = years of life lost.

### 3.5. Interpersonal violence attributed to smoking in male and female

The estimated burden attributed to smoking in men and women is shown in Tables 5–6. The number of DALY cases of interpersonal violence attributed to smoking was 130.88 per 1,00,000 males (95% uncertainty interval 94.33–170.85) in 2021. The number of DALY cases of interpersonal violence attributed to smoking was 12.09 per 1,00,000 in females (95% uncertainty interval 7.67–17.63) in 2021. Age-standardized DALYs rate per 1,00,000 in males was 0.27 (95% uncertainty interval 0.19–0.35), and in females was 0.03 (95% uncertainty interval 0.02–0.04) in 2021.

**Table 5:** Interpersonal violence (age-standardized DALYs, YLDs, YLLs, and deaths) attributed to smoking between 1990 and 2021 in male (per 1,00,000).

Measure	Metric	Age	Year					
			1990			2021		
			Value	Lower	Upper	Value	Lower	Upper
DALYs (disability-adjusted life years)	Number	All ages	57.1	40.54	76.65	130.88	94.33	170.85
	Percent	All ages	0.06	0.05	0.08	0.11	0.08	0.14
	Rate	All ages	0.2	0.14	0.26	0.3	0.22	0.39
	Percent	Age-standardized	0.11	0.08	0.14	0.1	0.08	0.13
	Rate	Age-standardized	0.35	0.25	0.47	0.27	0.19	0.35
YLDs (years lived with disability)	Number	All ages	21.64	14.2	31.42	47.6	31.52	68.57
	Percent	All ages	0.12	0.09	0.15	0.17	0.12	0.22
	Rate	All ages	0.07	0.05	0.11	0.11	0.07	0.16
	Percent	Age-standardized	0.17	0.13	0.22	0.17	0.13	0.23
	Rate	Age-standardized	0.14	0.09	0.2	0.1	0.07	0.15
YLLs (years of life lost)	Number	All ages	35.46	24.19	49.07	83.28	59.65	107.76
	Percent	All ages	0.05	0.04	0.07	0.09	0.07	0.12
	Rate	All ages	0.12	0.08	0.17	0.19	0.14	0.25
	Percent	Age-standardized	0.09	0.06	0.11	0.08	0.06	0.1
	Rate	Age-standardized	0.21	0.14	0.29	0.17	0.12	0.21
Deaths	Number	All ages	0.94	0.65	1.29	2.15	1.55	2.79
	Percent	All ages	0.08	0.06	0.11	0.13	0.1	0.16
	Rate	All ages	0	0	0	0	0	0.01
	Percent	Age-standardized	0.13	0.1	0.17	0.12	0.09	0.16
	Rate	Age-standardized	0.01	0	0.01	0	0	0.01

DALYs = disability-adjusted life years, YLDs = years lived with a disability, YLLs = years of life lost.

**Table 6:** Interpersonal violence (age-standardized DALYs, YLDs, YLLs, and deaths) attributed to smoking between 1990 and 2021 in female (per 1,00,000).

Measure	Metric	Year					
		1990			2021		
		Value	Lower	Upper	Value	Lower	Upper
DALYs (disability-adjusted life years)	Number	4.48	2.83	6.69	12.09	7.67	17.63
	Percent	0.01	0.01	0.02	0.03	0.02	0.04
	Rate	0.02	0.01	0.02	0.03	0.02	0.04
	Percent	0.02	0.02	0.04	0.03	0.02	0.04
	Rate	0.03	0.02	0.04	0.03	0.02	0.04
YLDs (years lived with disability)	Number	2.01	1.24	3.2	5.13	3.01	8.19
	Percent	0.01	0.01	0.02	0.02	0.01	0.04
	Rate	0.01	0	0.01	0.01	0.01	0.02
	Percent	0.03	0.01	0.04	0.02	0.01	0.04
	Rate	0.01	0.01	0.02	0.01	0.01	0.02
YLLs (years of life lost)	Number	2.47	1.53	3.67	6.96	4.37	10.04
	Percent	0.01	0.01	0.02	0.03	0.02	0.04
	Rate	0.01	0.01	0.01	0.02	0.01	0.02
	Percent	0.02	0.01	0.03	0.03	0.02	0.04
	Rate	0.02	0.01	0.02	0.01	0.01	0.02
Deaths	Number	0.07	0.04	0.1	0.2	0.13	0.28
	Percent	0.02	0.01	0.03	0.05	0.03	0.07
	Rate	0	0	0	0	0	0
	Percent	0.04	0.03	0.06	0.05	0.03	0.06
	Rate	0	0	0	0	0	0

DALYs = disability-adjusted life years, YLDs = years lived with a disability, YLLs = years of life lost.

### 3.6. Interpersonal violence attributed to smoking in provinces

**Figure 8.**
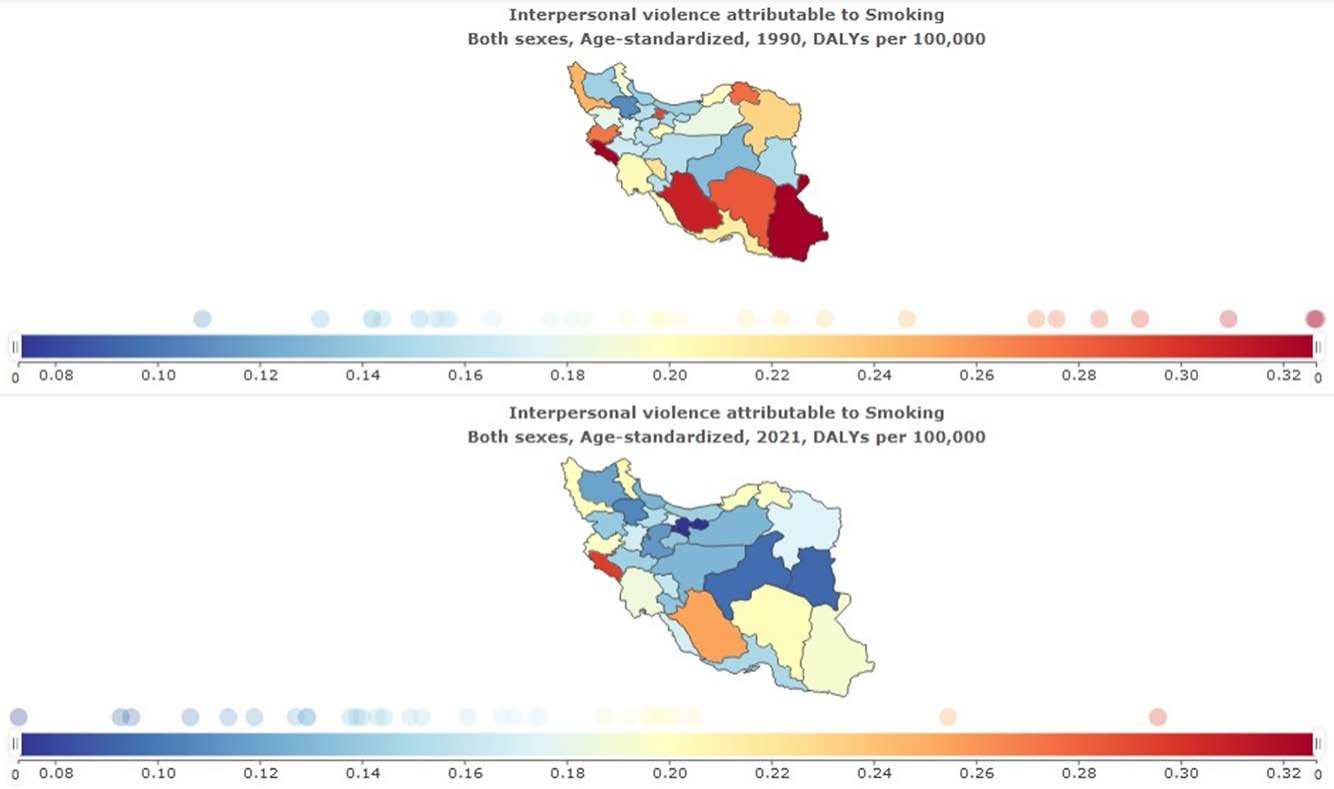
Interpersonal violence attributed to smoking in provinces.

The burden of interpersonal violence attributed to smoking in provinces is reported in Table 7 and Figure 8. The highest age-standardized DALYs rate per 1,00,000 in 2021 was for Ilam at 0.30 (95% uncertainty interval 0.20–0.40); the lowest was for Tehran at 0.07 (95% uncertainty interval 0.05–0.10).

**Table 7:** Interpersonal violence (age-standardized DALYs, YLDs, YLLs, and deaths) attributed to smoking between 1990 and 2021 in provinces (per 1,00,000).

Measure	Metric	Location	Age	Year					
				1990			2021		
				Value	Lower	Upper	Value	Lower	Upper
DALYs (disability-adjusted life years)	Number	Alborz	All ages	2.23	1.42	3.3	6.26	4.17	8.72
	Rate	Alborz	All ages	0.15	0.1	0.23	0.21	0.14	0.29
	Rate	Alborz	Age-standardized	0.29	0.19	0.42	0.17	0.12	0.24
	Number	Ardebil	All ages	1.17	0.75	1.69	3.12	2.12	4.38
	Rate	Ardebil	All ages	0.1	0.06	0.14	0.24	0.16	0.34
	Rate	Ardebil	Age-standardized	0.19	0.12	0.28	0.2	0.14	0.29
	Number	Bushehr	All ages	0.67	0.43	0.95	2.13	1.42	3.16
	Rate	Bushehr	All ages	0.09	0.06	0.14	0.17	0.11	0.25
	Rate	Bushehr	Age-standardized	0.2	0.13	0.28	0.17	0.11	0.25
	Number	Chahar Mahaal and Bakhtiari	All ages	0.78	0.52	1.15	1.72	1.15	2.44
	Rate	Chahar Mahaal and Bakhtiari	All ages	0.11	0.07	0.16	0.17	0.11	0.24
	Rate	Chahar Mahaal and Bakhtiari	Age-standardized	0.22	0.15	0.33	0.16	0.11	0.23
	Number	East Azarbayejan	All ages	2.94	2.02	4.28	5.97	4.07	8.27
	Rate	East Azarbayejan	All ages	0.09	0.06	0.12	0.14	0.1	0.2
	Rate	East Azarbayejan	Age-standardized	0.14	0.1	0.21	0.12	0.08	0.16
	Number	Fars	All ages	5.8	3.57	8.56	15.02	10.19	22.01
	Rate	Fars	All ages	0.16	0.1	0.24	0.29	0.2	0.43
	Rate	Fars	Age-standardized	0.31	0.19	0.46	0.25	0.17	0.36
	Number	Gilan	All ages	2.1	1.38	3.04	4.56	3.15	6.37
	Rate	Gilan	All ages	0.09	0.06	0.13	0.17	0.12	0.24
	Rate	Gilan	Age-standardized	0.14	0.09	0.21	0.13	0.09	0.18
	Number	Golestan	All ages	1.36	0.91	2.07	4.21	2.82	5.98
	Rate	Golestan	All ages	0.1	0.07	0.15	0.21	0.14	0.3
	Rate	Golestan	Age-standardized	0.2	0.13	0.3	0.2	0.13	0.28
	Number	Hamadan	All ages	1.67	1.11	2.35	3.58	2.29	5.15
	Rate	Hamadan	All ages	0.1	0.07	0.14	0.21	0.13	0.3
	Rate	Hamadan	Age-standardized	0.18	0.12	0.25	0.17	0.11	0.24
	Number	Hormozgan	All ages	0.94	0.62	1.35	2.6	1.72	3.72
	Rate	Hormozgan	All ages	0.1	0.07	0.15	0.13	0.09	0.19
	Rate	Hormozgan	Age-standardized	0.21	0.14	0.3	0.15	0.1	0.21
	Number	Ilam	All ages	0.64	0.42	0.93	2.07	1.4	2.88
	Rate	Ilam	All ages	0.15	0.1	0.21	0.34	0.23	0.47
	Rate	Ilam	Age-standardized	0.33	0.22	0.47	0.3	0.2	0.4
	Number	Isfahan	All ages	3.35	2.3	4.75	8.36	5.64	11.48
	Rate	Isfahan	All ages	0.09	0.06	0.13	0.16	0.1	0.21
	Rate	Isfahan	Age-standardized	0.16	0.11	0.22	0.13	0.09	0.18
	Number	Kerman	All ages	2.76	1.78	4.05	6.82	4.55	9.56
	Rate	Kerman	All ages	0.15	0.1	0.22	0.2	0.13	0.28
	Rate	Kerman	Age-standardized	0.28	0.18	0.41	0.2	0.14	0.28
	Number	Kermanshah	All ages	2.42	1.54	3.5	4.74	3.08	6.42
	Rate	Kermanshah	All ages	0.14	0.09	0.21	0.24	0.15	0.32
	Rate	Kermanshah	Age-standardized	0.27	0.18	0.4	0.2	0.13	0.27
	Number	Khorasan-e-Razavi	All ages	6.02	3.78	8.61	12.6	8.42	17.19
	Rate	Khorasan-e-Razavi	All ages	0.13	0.08	0.18	0.18	0.12	0.25
	Rate	Khorasan-e-Razavi	Age-standardized	0.23	0.14	0.33	0.17	0.12	0.23
	Number	Khuzestan	All ages	3.01	2.01	4.2	9.08	5.97	12.68
	Rate	Khuzestan	All ages	0.09	0.06	0.13	0.18	0.12	0.25
	Rate	Khuzestan	Age-standardized	0.2	0.14	0.28	0.19	0.12	0.26
	Number	Kohgiluyeh and Boyer-Ahmad	All ages	0.33	0.21	0.47	1.01	0.67	1.42
	Rate	Kohgiluyeh and Boyer-Ahmad	All ages	0.07	0.04	0.1	0.13	0.09	0.18
	Rate	Kohgiluyeh and Boyer-Ahmad	Age-standardized	0.16	0.1	0.23	0.14	0.09	0.19
	Number	Kurdistan	All ages	1.2	0.82	1.73	2.69	1.8	3.74
	Rate	Kurdistan	All ages	0.1	0.07	0.14	0.16	0.1	0.22
	Rate	Kurdistan	Age-standardized	0.18	0.13	0.26	0.14	0.09	0.19
	Number	Lorestan	All ages	1.24	0.81	1.89	2.88	1.93	4.17
	Rate	Lorestan	All ages	0.08	0.05	0.12	0.16	0.11	0.23
	Rate	Lorestan	Age-standardized	0.17	0.11	0.25	0.14	0.1	0.21
	Number	Markazi	All ages	1.11	0.73	1.6	2.05	1.35	2.96
	Rate	Markazi	All ages	0.09	0.06	0.13	0.14	0.09	0.2
	Rate	Markazi	Age-standardized	0.16	0.1	0.22	0.11	0.08	0.16
	Number	Mazandaran	All ages	2.08	1.38	3.01	6.45	4.41	8.86
	Rate	Mazandaran	All ages	0.08	0.05	0.12	0.18	0.12	0.25
	Rate	Mazandaran	Age-standardized	0.14	0.09	0.2	0.14	0.1	0.2
	Number	North Khorasan	All ages	0.97	0.66	1.38	1.81	1.24	2.52
	Rate	North Khorasan	All ages	0.16	0.1	0.22	0.21	0.14	0.29
	Rate	North Khorasan	Age-standardized	0.28	0.19	0.39	0.2	0.14	0.27
	Number	Qazvin	All ages	0.77	0.51	1.1	2.41	1.65	3.37
	Rate	Qazvin	All ages	0.08	0.05	0.12	0.18	0.12	0.25
	Rate	Qazvin	Age-standardized	0.15	0.1	0.22	0.15	0.11	0.21
	Number	Qom	All ages	0.73	0.48	1.1	2.08	1.41	2.96
	Rate	Qom	All ages	0.1	0.07	0.15	0.15	0.1	0.21
	Rate	Qom	Age-standardized	0.2	0.13	0.3	0.14	0.1	0.19
	Number	Semnan	All ages	0.54	0.36	0.77	1.1	0.74	1.56
	Rate	Semnan	All ages	0.12	0.08	0.16	0.14	0.1	0.2
	Rate	Semnan	Age-standardized	0.18	0.12	0.26	0.13	0.09	0.18
	Number	Sistan and Baluchistan	All ages	2.22	1.4	3.29	4	2.49	6.02
	Rate	Sistan and Baluchistan	All ages	0.15	0.1	0.22	0.13	0.08	0.19
	Rate	Sistan and Baluchistan	Age-standardized	0.33	0.21	0.48	0.19	0.13	0.28
	Number	South Khorasan	All ages	0.6	0.41	0.88	0.81	0.56	1.13
	Rate	South Khorasan	All ages	0.09	0.06	0.13	0.09	0.06	0.13
	Rate	South Khorasan	Age-standardized	0.15	0.1	0.22	0.09	0.06	0.13
	Number	Tehran	All ages	7.78	5.22	11.1	12.85	9.03	17.77
	Rate	Tehran	All ages	0.09	0.06	0.14	0.09	0.06	0.12
	Rate	Tehran	Age-standardized	0.15	0.1	0.21	0.07	0.05	0.1
	Number	West Azarbayejan	All ages	3.13	2.01	4.47	7.47	4.98	10.69
	Rate	West Azarbayejan	All ages	0.13	0.09	0.19	0.21	0.14	0.3
	Rate	West Azarbayejan	Age-standardized	0.25	0.16	0.35	0.2	0.13	0.28
	Number	Yazd	All ages	0.51	0.34	0.7	1.16	0.81	1.66
	Rate	Yazd	All ages	0.07	0.05	0.1	0.1	0.07	0.14
	Rate	Yazd	Age-standardized	0.13	0.09	0.18	0.09	0.07	0.13
	Number	Zanjan	All ages	0.52	0.35	0.75	1.34	0.92	1.84
	Rate	Zanjan	All ages	0.06	0.04	0.08	0.12	0.08	0.17
	Rate	Zanjan	Age-standardized	0.11	0.07	0.15	0.11	0.07	0.15
YLDs (years lived with disability)	Number	Alborz	All ages	0.85	0.55	1.24	2.45	1.56	3.61
	Rate	Alborz	All ages	0.06	0.04	0.09	0.08	0.05	0.12
	Rate	Alborz	Age-standardized	0.12	0.08	0.17	0.07	0.05	0.11
	Number	Ardebil	All ages	0.46	0.29	0.68	0.97	0.63	1.44
	Rate	Ardebil	All ages	0.04	0.03	0.06	0.07	0.05	0.11
	Rate	Ardebil	Age-standardized	0.08	0.05	0.12	0.07	0.04	0.1
	Number	Bushehr	All ages	0.26	0.17	0.39	0.72	0.46	1.06
	Rate	Bushehr	All ages	0.04	0.02	0.06	0.06	0.04	0.08
	Rate	Bushehr	Age-standardized	0.08	0.05	0.12	0.06	0.04	0.09
	Number	Chahar Mahaal and Bakhtiari	All ages	0.25	0.16	0.38	0.55	0.36	0.81
	Rate	Chahar Mahaal and Bakhtiari	All ages	0.03	0.02	0.05	0.05	0.04	0.08
	Rate	Chahar Mahaal and Bakhtiari	Age-standardized	0.07	0.05	0.11	0.05	0.04	0.08
	Number	East Azarbayejan	All ages	1.25	0.82	1.89	2.35	1.5	3.47
	Rate	East Azarbayejan	All ages	0.04	0.02	0.05	0.06	0.04	0.08
	Rate	East Azarbayejan	Age-standardized	0.06	0.04	0.1	0.05	0.03	0.07
	Number	Fars	All ages	1.72	1.09	2.51	4.5	2.91	6.64
	Rate	Fars	All ages	0.05	0.03	0.07	0.09	0.06	0.13
	Rate	Fars	Age-standardized	0.1	0.06	0.14	0.08	0.05	0.12
	Number	Gilan	All ages	0.75	0.47	1.11	1.82	1.15	2.69
	Rate	Gilan	All ages	0.03	0.02	0.05	0.07	0.04	0.1
	Rate	Gilan	Age-standardized	0.05	0.03	0.08	0.05	0.03	0.08
	Number	Golestan	All ages	0.46	0.29	0.66	1.3	0.84	1.92
	Rate	Golestan	All ages	0.03	0.02	0.05	0.07	0.04	0.1
	Rate	Golestan	Age-standardized	0.07	0.05	0.1	0.07	0.04	0.1
	Number	Hamadan	All ages	0.66	0.42	0.96	1.32	0.85	1.93
	Rate	Hamadan	All ages	0.04	0.03	0.06	0.08	0.05	0.11
	Rate	Hamadan	Age-standardized	0.07	0.05	0.11	0.06	0.04	0.09
	Number	Hormozgan	All ages	0.34	0.22	0.5	0.92	0.6	1.34
	Rate	Hormozgan	All ages	0.04	0.02	0.05	0.05	0.03	0.07
	Rate	Hormozgan	Age-standardized	0.08	0.05	0.12	0.06	0.04	0.08
	Number	Ilam	All ages	0.18	0.12	0.27	0.58	0.37	0.84
	Rate	Ilam	All ages	0.04	0.03	0.06	0.1	0.06	0.14
	Rate	Ilam	Age-standardized	0.1	0.06	0.14	0.09	0.06	0.13
	Number	Isfahan	All ages	1.49	0.96	2.17	3.5	2.24	5.1
	Rate	Isfahan	All ages	0.04	0.03	0.06	0.06	0.04	0.09
	Rate	Isfahan	Age-standardized	0.07	0.05	0.1	0.06	0.04	0.08
	Number	Kerman	All ages	0.85	0.55	1.25	2.07	1.34	3.04
	Rate	Kerman	All ages	0.05	0.03	0.07	0.06	0.04	0.09
	Rate	Kerman	Age-standardized	0.09	0.06	0.13	0.07	0.04	0.1
	Number	Kermanshah	All ages	0.76	0.47	1.11	1.57	1	2.28
	Rate	Kermanshah	All ages	0.04	0.03	0.07	0.08	0.05	0.11
	Rate	Kermanshah	Age-standardized	0.09	0.06	0.13	0.07	0.04	0.1
	Number	Khorasan-e-Razavi	All ages	2.03	1.29	3.06	4.28	2.69	6.3
	Rate	Khorasan-e-Razavi	All ages	0.04	0.03	0.06	0.06	0.04	0.09
	Rate	Khorasan-e-Razavi	Age-standardized	0.08	0.05	0.12	0.06	0.04	0.09
	Number	Khuzestan	All ages	1.05	0.68	1.59	2.98	1.93	4.33
	Rate	Khuzestan	All ages	0.03	0.02	0.05	0.06	0.04	0.09
	Rate	Khuzestan	Age-standardized	0.07	0.05	0.11	0.07	0.04	0.1
	Number	Kohgiluyeh and Boyer-Ahmad	All ages	0.14	0.09	0.21	0.42	0.27	0.63
	Rate	Kohgiluyeh and Boyer-Ahmad	All ages	0.03	0.02	0.04	0.05	0.03	0.08
	Rate	Kohgiluyeh and Boyer-Ahmad	Age-standardized	0.07	0.05	0.1	0.06	0.04	0.09
	Number	Kurdistan	All ages	0.46	0.3	0.67	1.08	0.7	1.61
	Rate	Kurdistan	All ages	0.04	0.02	0.05	0.06	0.04	0.09
	Rate	Kurdistan	Age-standardized	0.07	0.05	0.1	0.06	0.04	0.09
	Number	Lorestan	All ages	0.48	0.31	0.74	0.99	0.62	1.43
	Rate	Lorestan	All ages	0.03	0.02	0.05	0.06	0.03	0.08
	Rate	Lorestan	Age-standardized	0.07	0.04	0.1	0.05	0.03	0.08
	Number	Markazi	All ages	0.46	0.3	0.68	0.84	0.54	1.24
	Rate	Markazi	All ages	0.04	0.02	0.06	0.06	0.04	0.09
	Rate	Markazi	Age-standardized	0.07	0.04	0.1	0.05	0.03	0.07
	Number	Mazandaran	All ages	0.91	0.58	1.36	2.57	1.68	3.8
	Rate	Mazandaran	All ages	0.04	0.02	0.05	0.07	0.05	0.11
	Rate	Mazandaran	Age-standardized	0.06	0.04	0.1	0.06	0.04	0.09
	Number	North Khorasan	All ages	0.32	0.2	0.47	0.59	0.38	0.86
	Rate	North Khorasan	All ages	0.05	0.03	0.07	0.07	0.04	0.1
	Rate	North Khorasan	Age-standardized	0.1	0.06	0.14	0.07	0.04	0.1
	Number	Qazvin	All ages	0.3	0.19	0.45	0.81	0.51	1.22
	Rate	Qazvin	All ages	0.03	0.02	0.05	0.06	0.04	0.09
	Rate	Qazvin	Age-standardized	0.06	0.04	0.09	0.05	0.04	0.08
	Number	Qom	All ages	0.28	0.18	0.41	0.82	0.52	1.21
	Rate	Qom	All ages	0.04	0.03	0.06	0.06	0.04	0.08
	Rate	Qom	Age-standardized	0.08	0.05	0.12	0.06	0.04	0.09
	Number	Semnan	All ages	0.21	0.14	0.31	0.45	0.29	0.67
	Rate	Semnan	All ages	0.05	0.03	0.07	0.06	0.04	0.09
	Rate	Semnan	Age-standardized	0.07	0.05	0.11	0.05	0.04	0.08
	Number	Sistan and Baluchistan	All ages	0.73	0.47	1.07	1.18	0.76	1.73
	Rate	Sistan and Baluchistan	All ages	0.05	0.03	0.07	0.04	0.02	0.05
	Rate	Sistan and Baluchistan	Age-standardized	0.11	0.07	0.16	0.06	0.04	0.09
	Number	South Khorasan	All ages	0.35	0.22	0.51	0.38	0.25	0.56
	Rate	South Khorasan	All ages	0.05	0.03	0.08	0.04	0.03	0.06
	Rate	South Khorasan	Age-standardized	0.09	0.06	0.13	0.04	0.03	0.07
	Number	Tehran	All ages	4.14	2.68	6.08	7.15	4.74	10.64
	Rate	Tehran	All ages	0.05	0.03	0.07	0.05	0.03	0.07
	Rate	Tehran	Age-standardized	0.08	0.05	0.12	0.04	0.03	0.06
	Number	West Azarbayejan	All ages	1.09	0.71	1.62	2.51	1.62	3.71
	Rate	West Azarbayejan	All ages	0.05	0.03	0.07	0.07	0.05	0.11
	Rate	West Azarbayejan	Age-standardized	0.09	0.06	0.13	0.07	0.05	0.11
	Number	Yazd	All ages	0.23	0.15	0.35	0.53	0.34	0.78
	Rate	Yazd	All ages	0.03	0.02	0.05	0.04	0.03	0.06
	Rate	Yazd	Age-standardized	0.06	0.04	0.09	0.05	0.03	0.07
	Number	Zanjan	All ages	0.2	0.13	0.3	0.54	0.35	0.8
	Rate	Zanjan	All ages	0.02	0.01	0.03	0.05	0.03	0.07
	Rate	Zanjan	Age-standardized	0.04	0.03	0.07	0.04	0.03	0.07
YLLs (years of life lost)	Number	Alborz	All ages	1.38	0.76	2.21	3.8	2.21	5.66
	Rate	Alborz	All ages	0.1	0.05	0.15	0.13	0.07	0.19
	Rate	Alborz	Age-standardized	0.17	0.1	0.28	0.1	0.06	0.15
	Number	Ardebil	All ages	0.71	0.41	1.13	2.15	1.36	3.15
	Rate	Ardebil	All ages	0.06	0.04	0.1	0.17	0.1	0.24
	Rate	Ardebil	Age-standardized	0.11	0.07	0.18	0.14	0.09	0.2
	Number	Bushehr	All ages	0.41	0.24	0.63	1.42	0.9	2.27
	Rate	Bushehr	All ages	0.06	0.03	0.09	0.11	0.07	0.18
	Rate	Bushehr	Age-standardized	0.12	0.07	0.18	0.11	0.07	0.17
	Number	Chahar Mahaal and Bakhtiari	All ages	0.52	0.32	0.84	1.17	0.7	1.82
	Rate	Chahar Mahaal and Bakhtiari	All ages	0.07	0.04	0.12	0.11	0.07	0.18
	Rate	Chahar Mahaal and Bakhtiari	Age-standardized	0.15	0.09	0.24	0.11	0.06	0.16
	Number	East Azarbayejan	All ages	1.69	1.04	2.71	3.63	2.34	5.46
	Rate	East Azarbayejan	All ages	0.05	0.03	0.08	0.09	0.06	0.13
	Rate	East Azarbayejan	Age-standardized	0.08	0.05	0.13	0.07	0.04	0.1
	Number	Fars	All ages	4.08	2.27	6.45	10.53	6.41	16.32
	Rate	Fars	All ages	0.11	0.06	0.18	0.21	0.13	0.32
	Rate	Fars	Age-standardized	0.21	0.12	0.33	0.17	0.11	0.26
	Number	Gilan	All ages	1.35	0.82	2.15	2.75	1.75	3.99
	Rate	Gilan	All ages	0.06	0.04	0.09	0.11	0.07	0.15
	Rate	Gilan	Age-standardized	0.09	0.05	0.14	0.08	0.05	0.11
	Number	Golestan	All ages	0.9	0.53	1.49	2.91	1.84	4.23
	Rate	Golestan	All ages	0.07	0.04	0.11	0.15	0.09	0.21
	Rate	Golestan	Age-standardized	0.13	0.08	0.21	0.13	0.09	0.19
	Number	Hamadan	All ages	1.01	0.6	1.58	2.26	1.3	3.39
	Rate	Hamadan	All ages	0.06	0.04	0.09	0.13	0.07	0.19
	Rate	Hamadan	Age-standardized	0.1	0.06	0.16	0.1	0.06	0.15
	Number	Hormozgan	All ages	0.6	0.36	0.93	1.68	1.05	2.61
	Rate	Hormozgan	All ages	0.07	0.04	0.1	0.08	0.05	0.13
	Rate	Hormozgan	Age-standardized	0.14	0.08	0.21	0.09	0.06	0.14
	Number	Ilam	All ages	0.46	0.28	0.71	1.49	0.97	2.18
	Rate	Ilam	All ages	0.1	0.06	0.16	0.24	0.16	0.36
	Rate	Ilam	Age-standardized	0.23	0.14	0.35	0.2	0.14	0.3
	Number	Isfahan	All ages	1.86	1.12	2.88	4.86	3.03	7.32
	Rate	Isfahan	All ages	0.05	0.03	0.08	0.09	0.06	0.14
	Rate	Isfahan	Age-standardized	0.08	0.05	0.13	0.07	0.05	0.11
	Number	Kerman	All ages	1.91	1.14	3	4.75	2.96	6.98
	Rate	Kerman	All ages	0.1	0.06	0.16	0.14	0.09	0.2
	Rate	Kerman	Age-standardized	0.19	0.11	0.3	0.14	0.09	0.2
	Number	Kermanshah	All ages	1.66	0.97	2.57	3.16	1.91	4.6
	Rate	Kermanshah	All ages	0.1	0.06	0.15	0.16	0.1	0.23
	Rate	Kermanshah	Age-standardized	0.18	0.11	0.28	0.13	0.08	0.18
	Number	Khorasan-e-Razavi	All ages	3.99	2.4	6.29	8.32	5.23	12.14
	Rate	Khorasan-e-Razavi	All ages	0.08	0.05	0.13	0.12	0.08	0.18
	Rate	Khorasan-e-Razavi	Age-standardized	0.15	0.09	0.24	0.11	0.07	0.16
	Number	Khuzestan	All ages	1.96	1.17	2.93	6.1	3.77	9.13
	Rate	Khuzestan	All ages	0.06	0.04	0.09	0.12	0.08	0.18
	Rate	Khuzestan	Age-standardized	0.13	0.08	0.19	0.12	0.08	0.18
	Number	Kohgiluyeh and Boyer-Ahmad	All ages	0.19	0.1	0.29	0.59	0.35	0.89
	Rate	Kohgiluyeh and Boyer-Ahmad	All ages	0.04	0.02	0.06	0.08	0.05	0.11
	Rate	Kohgiluyeh and Boyer-Ahmad	Age-standardized	0.09	0.05	0.13	0.08	0.05	0.11
	Number	Kurdistan	All ages	0.74	0.47	1.16	1.61	0.99	2.35
	Rate	Kurdistan	All ages	0.06	0.04	0.09	0.09	0.06	0.14
	Rate	Kurdistan	Age-standardized	0.11	0.07	0.17	0.08	0.05	0.12
	Number	Lorestan	All ages	0.76	0.43	1.29	1.89	1.17	2.87
	Rate	Lorestan	All ages	0.05	0.03	0.08	0.11	0.07	0.16
	Rate	Lorestan	Age-standardized	0.1	0.06	0.17	0.09	0.06	0.14
	Number	Markazi	All ages	0.65	0.38	1.02	1.21	0.76	1.93
	Rate	Markazi	All ages	0.05	0.03	0.09	0.08	0.05	0.13
	Rate	Markazi	Age-standardized	0.09	0.05	0.14	0.07	0.04	0.1
	Number	Mazandaran	All ages	1.18	0.7	1.83	3.89	2.44	5.64
	Rate	Mazandaran	All ages	0.05	0.03	0.07	0.11	0.07	0.16
	Rate	Mazandaran	Age-standardized	0.08	0.05	0.12	0.08	0.05	0.12
	Number	North Khorasan	All ages	0.65	0.4	1.01	1.22	0.77	1.78
	Rate	North Khorasan	All ages	0.1	0.06	0.16	0.14	0.09	0.2
	Rate	North Khorasan	Age-standardized	0.18	0.11	0.28	0.13	0.08	0.19
	Number	Qazvin	All ages	0.47	0.28	0.71	1.6	0.99	2.34
	Rate	Qazvin	All ages	0.05	0.03	0.07	0.12	0.07	0.17
	Rate	Qazvin	Age-standardized	0.09	0.06	0.14	0.1	0.06	0.14
	Number	Qom	All ages	0.46	0.27	0.75	1.26	0.79	1.98
	Rate	Qom	All ages	0.06	0.04	0.1	0.09	0.06	0.14
	Rate	Qom	Age-standardized	0.12	0.07	0.2	0.08	0.05	0.12
	Number	Semnan	All ages	0.33	0.2	0.51	0.66	0.42	0.99
	Rate	Semnan	All ages	0.07	0.04	0.11	0.08	0.05	0.13
	Rate	Semnan	Age-standardized	0.11	0.07	0.17	0.07	0.05	0.11
	Number	Sistan and Baluchistan	All ages	1.49	0.86	2.42	2.82	1.58	4.76
	Rate	Sistan and Baluchistan	All ages	0.1	0.06	0.17	0.09	0.05	0.15
	Rate	Sistan and Baluchistan	Age-standardized	0.21	0.12	0.34	0.13	0.08	0.21
	Number	South Khorasan	All ages	0.26	0.15	0.42	0.44	0.27	0.67
	Rate	South Khorasan	All ages	0.04	0.02	0.06	0.05	0.03	0.08
	Rate	South Khorasan	Age-standardized	0.06	0.04	0.1	0.05	0.03	0.07
	Number	Tehran	All ages	3.64	2.06	5.81	5.69	3.7	8.59
	Rate	Tehran	All ages	0.04	0.03	0.07	0.04	0.03	0.06
	Rate	Tehran	Age-standardized	0.07	0.04	0.11	0.03	0.02	0.05
	Number	West Azarbayejan	All ages	2.04	1.16	3.11	4.96	3.04	7.37
	Rate	West Azarbayejan	All ages	0.09	0.05	0.13	0.14	0.09	0.21
	Rate	West Azarbayejan	Age-standardized	0.16	0.09	0.24	0.13	0.08	0.19
	Number	Yazd	All ages	0.28	0.17	0.41	0.63	0.37	0.95
	Rate	Yazd	All ages	0.04	0.02	0.06	0.05	0.03	0.08
	Rate	Yazd	Age-standardized	0.07	0.04	0.1	0.05	0.03	0.07
	Number	Zanjan	All ages	0.32	0.19	0.48	0.8	0.51	1.2
	Rate	Zanjan	All ages	0.04	0.02	0.05	0.07	0.05	0.11
	Rate	Zanjan	Age-standardized	0.07	0.04	0.1	0.06	0.04	0.09
Deaths	Number	Alborz	All ages	0.04	0.02	0.06	0.1	0.06	0.15
	Rate	Alborz	All ages	0	0	0	0	0	0
	Rate	Alborz	Age-standardized	0	0	0.01	0	0	0
	Number	Ardebil	All ages	0.02	0.01	0.03	0.06	0.04	0.08
	Rate	Ardebil	All ages	0	0	0	0	0	0.01
	Rate	Ardebil	Age-standardized	0	0	0	0	0	0.01
	Number	Bushehr	All ages	0.01	0.01	0.02	0.04	0.02	0.06
	Rate	Bushehr	All ages	0	0	0	0	0	0
	Rate	Bushehr	Age-standardized	0	0	0	0	0	0
	Number	Chahar Mahaal and Bakhtiari	All ages	0.01	0.01	0.02	0.03	0.02	0.05
	Rate	Chahar Mahaal and Bakhtiari	All ages	0	0	0	0	0	0
	Rate	Chahar Mahaal and Bakhtiari	Age-standardized	0	0	0.01	0	0	0
	Number	East Azarbayejan	All ages	0.05	0.03	0.07	0.09	0.06	0.14
	Rate	East Azarbayejan	All ages	0	0	0	0	0	0
	Rate	East Azarbayejan	Age-standardized	0	0	0	0	0	0
	Number	Fars	All ages	0.11	0.06	0.17	0.27	0.17	0.42
	Rate	Fars	All ages	0	0	0	0.01	0	0.01
	Rate	Fars	Age-standardized	0.01	0	0.01	0	0	0.01
	Number	Gilan	All ages	0.04	0.02	0.06	0.07	0.05	0.1
	Rate	Gilan	All ages	0	0	0	0	0	0
	Rate	Gilan	Age-standardized	0	0	0	0	0	0
	Number	Golestan	All ages	0.02	0.01	0.04	0.08	0.05	0.11
	Rate	Golestan	All ages	0	0	0	0	0	0.01
	Rate	Golestan	Age-standardized	0	0	0.01	0	0	0.01
	Number	Hamadan	All ages	0.03	0.02	0.04	0.06	0.03	0.09
	Rate	Hamadan	All ages	0	0	0	0	0	0
	Rate	Hamadan	Age-standardized	0	0	0	0	0	0
	Number	Hormozgan	All ages	0.02	0.01	0.02	0.04	0.03	0.06
	Rate	Hormozgan	All ages	0	0	0	0	0	0
	Rate	Hormozgan	Age-standardized	0	0	0.01	0	0	0
	Number	Ilam	All ages	0.01	0.01	0.02	0.04	0.03	0.06
	Rate	Ilam	All ages	0	0	0	0.01	0	0.01
	Rate	Ilam	Age-standardized	0.01	0	0.01	0.01	0	0.01
	Number	Isfahan	All ages	0.05	0.03	0.08	0.13	0.08	0.19
	Rate	Isfahan	All ages	0	0	0	0	0	0
	Rate	Isfahan	Age-standardized	0	0	0	0	0	0
	Number	Kerman	All ages	0.05	0.03	0.08	0.12	0.08	0.18
	Rate	Kerman	All ages	0	0	0	0	0	0.01
	Rate	Kerman	Age-standardized	0.01	0	0.01	0	0	0.01
	Number	Kermanshah	All ages	0.04	0.03	0.07	0.08	0.05	0.12
	Rate	Kermanshah	All ages	0	0	0	0	0	0.01
	Rate	Kermanshah	Age-standardized	0	0	0.01	0	0	0
	Number	Khorasan-e-Razavi	All ages	0.11	0.06	0.17	0.21	0.14	0.31
	Rate	Khorasan-e-Razavi	All ages	0	0	0	0	0	0
	Rate	Khorasan-e-Razavi	Age-standardized	0	0	0.01	0	0	0
	Number	Khuzestan	All ages	0.05	0.03	0.08	0.16	0.1	0.23
	Rate	Khuzestan	All ages	0	0	0	0	0	0
	Rate	Khuzestan	Age-standardized	0	0	0.01	0	0	0
	Number	Kohgiluyeh and Boyer-Ahmad	All ages	0	0	0.01	0.02	0.01	0.02
	Rate	Kohgiluyeh and Boyer-Ahmad	All ages	0	0	0	0	0	0
	Rate	Kohgiluyeh and Boyer-Ahmad	Age-standardized	0	0	0	0	0	0
	Number	Kurdistan	All ages	0.02	0.01	0.03	0.04	0.03	0.06
	Rate	Kurdistan	All ages	0	0	0	0	0	0
	Rate	Kurdistan	Age-standardized	0	0	0	0	0	0
	Number	Lorestan	All ages	0.02	0.01	0.03	0.05	0.03	0.07
	Rate	Lorestan	All ages	0	0	0	0	0	0
	Rate	Lorestan	Age-standardized	0	0	0	0	0	0
	Number	Markazi	All ages	0.02	0.01	0.03	0.03	0.02	0.05
	Rate	Markazi	All ages	0	0	0	0	0	0
	Rate	Markazi	Age-standardized	0	0	0	0	0	0
	Number	Mazandaran	All ages	0.03	0.02	0.05	0.1	0.07	0.15
	Rate	Mazandaran	All ages	0	0	0	0	0	0
	Rate	Mazandaran	Age-standardized	0	0	0	0	0	0
	Number	North Khorasan	All ages	0.02	0.01	0.03	0.03	0.02	0.04
	Rate	North Khorasan	All ages	0	0	0	0	0	0.01
	Rate	North Khorasan	Age-standardized	0	0	0.01	0	0	0
	Number	Qazvin	All ages	0.01	0.01	0.02	0.04	0.03	0.06
	Rate	Qazvin	All ages	0	0	0	0	0	0
	Rate	Qazvin	Age-standardized	0	0	0	0	0	0
	Number	Qom	All ages	0.01	0.01	0.02	0.03	0.02	0.05
	Rate	Qom	All ages	0	0	0	0	0	0
	Rate	Qom	Age-standardized	0	0	0.01	0	0	0
	Number	Semnan	All ages	0.01	0.01	0.01	0.02	0.01	0.03
	Rate	Semnan	All ages	0	0	0	0	0	0
	Rate	Semnan	Age-standardized	0	0	0	0	0	0
	Number	Sistan and Baluchistan	All ages	0.04	0.02	0.06	0.07	0.04	0.12
	Rate	Sistan and Baluchistan	All ages	0	0	0	0	0	0
	Rate	Sistan and Baluchistan	Age-standardized	0.01	0	0.01	0	0	0.01
	Number	South Khorasan	All ages	0.01	0	0.01	0.01	0.01	0.02
	Rate	South Khorasan	All ages	0	0	0	0	0	0
	Rate	South Khorasan	Age-standardized	0	0	0	0	0	0
	Number	Tehran	All ages	0.09	0.05	0.15	0.15	0.1	0.22
	Rate	Tehran	All ages	0	0	0	0	0	0
	Rate	Tehran	Age-standardized	0	0	0	0	0	0
	Number	West Azarbayejan	All ages	0.05	0.03	0.08	0.13	0.08	0.19
	Rate	West Azarbayejan	All ages	0	0	0	0	0	0.01
	Rate	West Azarbayejan	Age-standardized	0	0	0.01	0	0	0.01
	Number	Yazd	All ages	0.01	0	0.01	0.02	0.01	0.02
	Rate	Yazd	All ages	0	0	0	0	0	0
	Rate	Yazd	Age-standardized	0	0	0	0	0	0
	Number	Zanjan	All ages	0.01	0.01	0.01	0.02	0.01	0.03
	Rate	Zanjan	All ages	0	0	0	0	0	0
	Rate	Zanjan	Age-standardized	0	0	0	0	0	0

DALYs = disability-adjusted life years, YLDs = years lived with a disability, YLLs = years of life lost.

## 4. Discussion

This research was conducted to investigate the burden of interpersonal violence attributed to smoking in Iran and also a comparison was made between Iran and Global. This study was based on the Global Burden of Disease 2021.^[30–32]^ Summary exposure value for smoking, DALYs, YLDs, YLLs, and death of interpersonal violence attributed to smoking were evaluated.

The result showed that the burden caused by smoking in Iran and the world has decreased compared to 1990. As it was shown in the previous estimates, there was a trend in the reduction of smoking in the world as well as in Iran.^[3]^ Also, the examination of smoking rates in men and women compared to previous estimates^[3]^ showed that men are still more exposed to the burden of smoking compared to women, and the prevalence of smoking is much higher in men. Health policies related to smoking control can play a role in this reduction, although the beginning of the smoking control policy dates back to 1960, which started in some countries, but with the determination of the effects of smoking, global progress was made in smoking control.^[3,35,36]^ After the start of the World Health Organization framework convention on tobacco control a rapid decline in smoking prevalence occurred in many countries.^[37]^ While the observed decline in cigarette smoking is encouraging, it is important to consider whether this reduction might be offset by the rise in alternative forms of tobacco use, such as e-cigarettes and shisha.^[38,39]^ These products have gained popularity in recent years, particularly among younger populations, and may serve as substitutes for traditional cigarettes. However, their long-term health impacts and contributions to behaviors like interpersonal violence remain under-researched. Future studies should investigate the prevalence and effects of these alternative tobacco products to provide a comprehensive understanding of their role in public health and potential implications for policy.

The burden of interpersonal violence attributed to smoking was another finding that was investigated in this study. The result showed that the burden of interpersonal violence attributed to smoking has decreased significantly in the last 3 decades at the global level, this decrease can also be seen in Iran, however, at the global level, the rate of decrease has been higher, while that there has been less fluctuation in Iran during the decades. There are mechanisms involved in how smoking is related to interpersonal violence.^[22]^ A mechanism refers to the neurobiological mechanism. Nicotine and tobacco components affect brain regions and brain circuits associated with aggression, and psychological states such as anxiety, impulsivity, and mood.^[40–43]^ Evidence from human studies also suggests an association between nicotine, hostility, and aggression.^[28,44]^ It is also necessary to note that interpersonal violence may be a risk factor for smoking initiation.^[29,45]^ In other words, people who are exposed to violence may use smoking as a way to relieve themselves.^[46]^

## 5. Strengths and limitations

Studying also has several strengths. It leverages the robust Global Burden of Disease 2021 dataset, providing a comprehensive and standardized assessment of the burden of interpersonal violence attributed to smoking. This study is among the first to quantify this relationship in Iran, offering valuable insights for policymakers. Even though the effects of smoking on health have been extensively studied, the studies on smoking and its effects on interpersonal violence are very few, and the previous studies are very rare. This study was able to add to the scientific literature on interpersonal violence attributed to smoking. Nevertheless, the study has several methodological limitations that should be considered when interpreting the results. First the study design relies on related to the Global Burden of Disease 2021,^[30–32]^ which may have inherent limitations in data collection and reporting across different regions. Second, the study did not account for all potential confounding variables, such as socioeconomic status or psychological factors, which could influence both smoking behavior and interpersonal violence. Third, the study focused primarily on cigarette smoking and did not include other forms of tobacco use, such as shisha or e-cigarettes, which may have different effects on violent behavior. Despite these limitations, the observed decline in smoking prevalence and its associated burden of interpersonal violence suggests that public health interventions may be effective. However, the lack of data on alternative tobacco products and potential confounders means that the full extent of the relationship between smoking and interpersonal violence may not be fully captured. Future research should address these gaps to provide a more comprehensive understanding. In future studies, it is necessary to pay attention to cohort studies on the relationship between smoking and interpersonal violence. This study cannot demonstrate a causal relationship between cigarette smoking and interpersonal violence, and this should be taken into account when interpreting the relationships.

The findings have broad implications for the scientific community and public health practice. They highlight the need for integrated policies that address both smoking cessation and violence prevention. The results suggest that reducing smoking prevalence may have benefits beyond physical health, potentially reducing interpersonal violence. These insights can inform global and national health strategies, particularly in regions with high smoking rates and violence burden. Future research should explore the mechanisms linking smoking to violence and evaluate the effectiveness of combined interventions.

## 6. Conclusion

The summary of this research shows that the burden of interpersonal violence attributed to smoking is significant both at the global and national levels, although the prevalence of smoking has decreased in recent decades, it is necessary to implement health policies in order to reduce the damage caused by smoking as much as possible, it will be beneficial to increase the awareness of the general population.

## Acknowledgments

The data sources of this study were taken from GBD 2021, which is publicly available.

## Author contributions

**Conceptualization:** Moien A.B. Khan, Sohrab Amiri.

**Visualization:** Moien A.B. Khan.

**Writing – original draft:** Moien A.B. Khan, Sohrab Amiri.

**Writing – review & editing:** Moien A.B. Khan, Sohrab Amiri.
